# Is Time-Restricted Eating Safe in the Treatment of Type 2 Diabetes?—A Review of Intervention Studies

**DOI:** 10.3390/nu14112299

**Published:** 2022-05-30

**Authors:** Sarah Uldal, Kim Katrine Bjerring Clemmensen, Frederik Persson, Kristine Færch, Jonas Salling Quist

**Affiliations:** 1 Clinical Research, Copenhagen University Hospital—Steno Diabetes Center Copenhagen, DK-2730 Herlev, Denmark; sarah.uldal@gmail.com (S.U.); kim.katrine.bjerring.clemmensen.01@regionh.dk (K.K.B.C.); frederik.persson@regionh.dk (F.P.); kristine.faerch@regionh.dk (K.F.); 2 Novo Nordisk A/S, DK-2860 Søborg, Denmark; 3 Department of Biomedical Sciences, Faculty of Health and Medical Sciences, University of Copenhagen, DK-2200 Copenhagen, Denmark; 4 Appetite Control and Energy Balance Research Group, School of Psychology, Faculty of Medicine & Health, University of Leeds, Leeds LS2 9JT, UK

**Keywords:** type 2 diabetes, overweight, obesity, intermittent fasting, time-restricted eating, safety, hypoglycaemia, weight loss, intervention studies

## Abstract

Time-restricted eating (TRE) has been shown to improve body weight and glucose metabolism in people at high risk of type 2 diabetes. However, the safety of TRE in the treatment of type 2 diabetes is unclear. We investigated the safety of TRE interventions in people with type 2 diabetes by identifying published and ongoing studies. Moreover, we identified the commonly used antidiabetic drugs and discussed the safety of TRE in people with type 2 diabetes considering the use of these drugs. In addition, we addressed the research needed before TRE can be recommended in the treatment of type 2 diabetes. A literature search was conducted to identify published (MEDLINE PubMed) and ongoing studies (ClinicalTrials.gov) on TRE in people with type 2 diabetes. To assess the usage of antidiabetic drugs and to discuss pharmacodynamics and pharmacokinetics in a TRE context, the most used antidiabetic drugs were identified and analysed. Statistics regarding sale of pharmaceuticals were obtained from MEDSTAT.DK which are based on data from the national Register of Medicinal Product Statistics, and from published studies on medication use in different countries. Four published studies investigating TRE in people with type 2 diabetes were identified as well as 14 ongoing studies. The completed studies suggested that TRE is safe among people with type 2 diabetes. Common antidiabetic drugs between 2010 and 2019 were metformin, insulin, dipeptidyl peptidase-4 inhibitors, glucagon-like peptide-1 receptor agonists, sulfonylureas, and sodium-glucose cotransporter-2 inhibitors. Existing studies suggest that TRE is not associated with major safety issues in people with type 2 diabetes as long as medication is monitored and adjusted. However, because of low generalisability of the few studies available, more studies are needed to make concrete recommendations regarding efficacy and safety of TRE in people with type 2 diabetes.

## 1. Introduction

The prevalence of type 2 diabetes has increased along with the rising incidence of obesity, and high body mass index (BMI), as a measure for excess adiposity, is a major risk factor for type 2 diabetes [1,2]. Both the American Diabetes Association (ADA) and European Association for the Study of Diabetes (EASD) recommend changes in lifestyle as the first step in prevention and treatment of type 2 diabetes [3]. However, it is difficult to obtain and maintain weight loss in the long term [4]. Thus, there is a need for novel and feasible approaches offering simple and effective ways to obtain and maintain weight loss. 

Today, the most common strategies for weight loss are calorie-restricted diets [5,6]. This approach is effective; however, many people find it difficult to adhere to this type of dieting [5]. An alternative to calorie restriction is intermittent fasting which covers weight loss regimens alternating between eating and defined periods of prolonged fasting [5]. Intermittent fasting regimens can be grouped into different categories; one of these being time-restricted eating (TRE), which has been suggested as a feasible and effective lifestyle intervention and has recently become increasingly popular, which may be due to its simple nature and the intervention not being restrictive in terms of calorie intake and diet composition.

Time-restricted feeding in animals and TRE in humans limits food intake to a period, typically less than 12 h per day during day time (active phase in animals), thereby extending the time spent in the fasted state [7]. The underlying theory and goal of many TRE regimens are to align the cycle of feeding and fasting with the circadian rhythms [8], which are circadian oscillations in metabolic pathways [9]. The suprachiasmatic nucleus of the hypothalamus serves as a master clock in regulating peripheral systems and is mainly synchronised by light exposure [9,10]. Clocks are also present in peripheral tissues and these are affected by the feeding/fasting cycles [10]. Internal desynchronisation caused by irregular feeding has been shown to decrease insulin sensitivity and cause postprandial glucose intolerance [10,11]. This supports the theory that TRE may lead to improvement in metabolic health regardless of weight loss [12]. Studies have shown that people tend to spontaneously reduce their caloric intake by 7–22% when following a TRE regimen with ad libitum food intake [7]. Recent studies have shown that TRE can lead to weight loss and improvement of insulin sensitivity and glucose tolerance in people with overweight and high risk of type 2 diabetes [7,13]. This indicates that TRE could be a promising approach to obtain weight loss and improve glycaemic control in people with type 2 diabetes. In an opinion paper [14], concerns regarding the lack of evidence for the safety and health effects in people with type 2 diabetes engaging in intermittent fasting were addressed. One of the main concerns is the risk of hypoglycaemia due to concomitant treatment with antidiabetic drugs [14]. It was concluded that a more thorough evaluation of medication safety is needed before suggesting that people with type 2 diabetes should engage in intermittent fasting regimens [14]. 

The aim of this review was therefore to investigate the safety of TRE interventions in people with type 2 diabetes treated with antidiabetic drugs. Specific objectives were: (1) to identify published and ongoing studies in this area and to assess the safety of TRE in these studies; (2) to identify the commonly used antidiabetic drugs and discuss the safety of TRE in people with type 2 diabetes considering the use of these antidiabetic drugs; (3) to discuss which research is needed before TRE can be recommended in the treatment of type 2 diabetes.

## 2. Materials and Methods

### 2.1. Search Strategy for Identifying Studies Investigating TRE in People with Type 2 Diabetes

A literature search in MEDLINE PubMed was performed on 17 November 2021 using two blocks of search strings. Owing to the exploratory nature of the review, literature search was only performed in one database. The first block describes the population of interest (people with type 2 diabetes) and was created by combining the MeSH Term [15] for type 2 diabetes and text words with OR in PubMed. The second block was describing the intervention (time-restricted eating) and did not include any MeSH Terms as the closest relevant MeSH Term was fasting. Using this term would make the search too broad. The two blocks were put together resulting in the following search string: ((“Type 2 Diabetes” OR “Diabetes type 2” OR “type two diabetes” OR “Type 2 Diabetic” OR “T2D” OR “Diabetic type 2” OR “Non-Insulin-Dependent” OR “NIDDM”) OR (“Diabetes Mellitus, Type 2”[Mesh])) AND ((“intermittent fasting”) OR ((“time restricted feeding”) OR (“time restricted eating”))). The search gave a total of 110 hits. No duplicates were detected. The screening process was divided into two steps [16]. In the first step, the articles were evaluated based on their title and abstract checking for the following inclusion criteria: (1) TRE intervention; (2) study population of people with type 2 diabetes; (3) English language. In the second step, the articles were assessed based on full-text screening to see if they reported on the risk of hypoglycaemia or other adverse events.

### 2.2. Ongoing Studies of TRE in People with Type 2 Diabetes

To identify ongoing studies on TRE in people with type 2 diabetes, a search was conducted on 21 December 2021 using the search phrase “time restricted feeding OR time restricted eating” in the search field “other terms” on ClinicalTrials.gov. A criterion for including the studies was that they should have an inclusion criterion of elevated blood glucose levels or type 2 diabetes and no exclusion criteria of type 2 diabetes.

### 2.3. Selection and Description of Antidiabetic Drugs

A description of commonly used antidiabetic drugs is included in this paper to address the potential of TRE affecting effectiveness and safety of antidiabetic drugs in people with type 2 diabetes. The most used antidiabetic drugs were identified by examining the sales data from the medicinal product statistics [15] and from reports from different countries identified through a search in the PubMed database. The pharmacodynamic and pharmacokinetic profiles of the most used classes of antidiabetic drugs identified are described briefly. As different drugs within the same class of drugs can vary in pharmacodynamic and pharmacokinetic profile, the main mechanisms of the classes are described. The description is based on reviews of antidiabetic drugs identified through a search in the PubMed database.

### 2.4. Statistics on Antidiabetic Drugs

Statistics regarding sale of pharmaceuticals in Denmark are available on the webpage MEDSTAT.DK. The statistics are based on data from the Register of Medicinal Product Statistics [15]. The register includes data for all years since 1996.The search module “ATC code” was chosen in order to find the total sales of all antidiabetic drugs in Denmark. In the search module “ATC code” the anatomical group A (Alimentary tract and metabolism) was chosen and subsequently group 10 (Drugs used in diabetes) was marked. All categories within group A10 were extracted to collect data on the individual antidiabetic drugs. For this review, data were retrieved for the entire country in order to identify the most used antidiabetic drugs [15]. Data from the last 10 years for the primary sector were extracted as the number of users per 1000 inhabitants. A person counts as a user if he or she has bought prescription medicine within the relevant year [15]. Approximately 99% of the total sales were prescription sales for these ATC codes and years [15]. The figure was made in R studio (R version 3.6.3. and R studio version 1.2.5042).

## 3. Results

### 3.1. Time-Restricted Eating Interventions in People with Type 2 Diabetes

Table 1 summarises the four studies investigating TRE in people with type 2 diabetes identified through the literature search [17,18,19,20].

The study by Arnason et al. [19] aimed to determine biochemical effects and clinical tolerability. Regarding safety, the study concluded that intermittent fasting may be tolerable and safe. The study was a small pilot study with 10 participants and a 2-week intervention. The participants were educated on the risk of hypoglycaemia and how to detect and manage it. People with hypoglycaemic unawareness and use of insulin or glyburide were excluded. Morning, afternoon, and evening blood glucose levels were measured daily throughout the 2-week intervention. The fasting goal was 18–20 h, but participants did not reach this goal. However, participants increased the time spent in the fasted state from a baseline of 11.6 h to 16.8 h during the intervention. There were no incidences of hypoglycaemia. The study did not report on adverse events [19].

In a cross-over study, Kahleova et al. [18] investigated the effect of six meals (breakfast, lunch, and dinner and three snacks) a day compared to two meals (breakfast: 6–10 a.m. and lunch: 12–4 p.m.) a day with a similar calorie restriction (500 kcal/day) on body weight, hepatic fat content, insulin resistance, and beta cell function. The study suggested that two meals a day might be more beneficial than six meals a day for people with type 2 diabetes. The study did not have a primary outcome investigating safety or tolerability and did not report the specific timing of meals. Participants were instructed to continue their antidiabetic medication regimen unless they experienced repeated occurrences of hypoglycaemia. A glucometer and instructions on how to use it were given to all participants. The study did not report on hypoglycaemic events. However, no adverse events were registered [18].

The study by Parr et al. [17] had feasibility as primary outcome. One of their indicators of feasibility was safety. They excluded potential participants if they were taking more than two oral antidiabetic drugs or if they were taking sulfonylureas, insulin, or glucagon-like peptite-1 receptor agonists (GLP-1-RAs). The participants did not report any incidences of hypoglycaemia [17]. However, the study did not mention any instructions to measure blood glucose levels. The participants were asked to eat between 10 a.m. and 7 p.m. on as many days as possible during the 4-week TRE intervention. The study concluded that the eating regimen was feasible for at least 5 days per week. No adverse events were reported [17]. 

In a recent randomised controlled trial (RCT), Che et al. [20] investigated effects of 12 weeks 10 h TRE (8 a.m.–6 p.m.) in 120 people with overweight and type 2 diabetes. Approximately 70% of the participants used oral antidiabetic drugs (unspecified) and approximately 30% used insulin (unspecified) and they had no exclusion criteria regarding antidiabetic medication. Among several outcomes, they observed weight loss and a reduction in HbA_1c_ and use of antidiabetic medication, and no adverse or hypoglycaemic events in response to TRE [20].

In addition to the four published studies, the search on ClinicalTrials.gov resulted in 119 hits. Fifteen of these studies met the selected inclusion and exclusion criteria. One study did not describe a daily eating window and was therefore excluded. Thus, a total of 14 ongoing or completed, yet not reported, studies investigating TRE in people with type 2 diabetes were identified. Table 2 gives an overview of the relevant trials registered at ClinicalTrials.gov on 21 December 2021.

### 3.2. Medication Used for Treatment of Type 2 Diabetes

When people are diagnosed with type 2 diabetes, they are advised about diabetes management, as well as diet and exercise and they are offered help to quit smoking [21]. In addition to lifestyle intervention, treatment with antidiabetic drugs is initiated. The first choice of drug is metformins [22]. If the person with type 2 diabetes does not tolerate metformin, or if an acceptable HbA_1c_ level is not obtained on metformin and lifestyle changes alone, a drug from the second line of treatment is chosen. The drugs listed as the second line of treatment are: dipeptidyl peptidase-4 inhibitors (DPP-4 inhibitors), sulfonylureas, sodium-glucose cotransporter-2 (SGLT-2) inhibitors, GLP-1-RAs, or basal insulins. It is recommended to individualise the decision on which drug to give based on comorbidity (e.g., cardiovascular disease or chronic kidney disease) and on individual preferences. If acceptable HbA_1c_ levels are still not obtained with the second line of treatment, the third line of treatment can be pursued. Insulin treatment can be intensified with use of rapid-acting insulin or premixed insulin [22,23].

### 3.3. Most Used Antidiabetic Drugs

**Figure 1:**
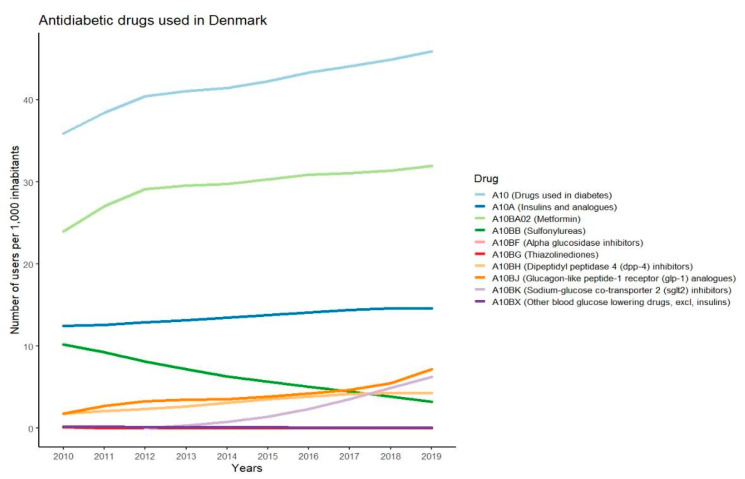
Graphs showing number of users per 1000 inhabitants in Denmark for different antidiabetic drugs in the years 2010–2019.

The changes in users per 1000 inhabitants during the last 10 years for the different classes of antidiabetic drugs used in Denmark are presented in Figure 1. The data show that metformin is the most used antidiabetic drug, with more than 30 users per 1000 inhabitants. Insulin is the second most used drug and the number of users has slightly increased over the past years. The use of sulfonylureas has declined while the use of GLP-1-RAs and SGLT-2 inhibitors has increased. The use of DPP-4 inhibitors has been at a constant level for the past few years and alpha glucosidase inhibitors, thiazolidinediones, and other blood-glucose-lowering drugs within the A10BX group are at a low to non-existing level of use in Denmark. The antidiabetic drugs chosen for further description in this paper are metformin, sulfonylureas, DPP-4 inhibitors, GLP-1-RAs, SGLT-2 inhibitors, and basal insulins. These drugs are chosen since they are recommended in the diabetes guidelines and several reports show that they are the most frequently prescribed drugs in countries such as Sweden, Norway, Australia, the United Kingdom, and Canada [24,25,26,27].

#### 3.3.1. Metformin

Metformin is a biguanide approved for use in Europe since 1957 [28,29]. Despite its long-term use, many details of the actions of metformin are still to be determined [28]. In the setting of type 2 diabetes, the main mechanism is inhibiting gluconeogenesis in the liver, resulting in a reduced hepatic glucose output [28]. Other reported effects of metformin include increased glucose utilisation in the intestine, stimulation of GLP-1 secretion, ability to alter the gut microbiome, and activation of AMP-activated protein kinase, which is thought to play a role in the effects of metformin on fatty acid oxidation and insulin sensitivity [28,29]. The bioavailability of metformin is 40–60% and it is not metabolised in the body but excreted unchanged in the urine [29]. Approximately three hours after the administration of the drug, the peak plasma concentration is reached [29]. It is recommended to take metformin twice daily with meals in order to minimise the risk of gastrointestinal side effects, which 10–15% of users experience [23]. The only known safety concern for metformin is that it causes subclinical increases in lactic acid. Ingesting a large overdose thus seems to cause lactic acidosis [30]. There seems to be no risk of hypoglycaemia associated with metformin use, and risk of metformin use in combination with TRE is anticipated to be low [30].

#### 3.3.2. GLP-1 Receptor Agonists

GLP-1-RAs activate the GLP-1 receptor (GLP-1R) by mimicking GLP-1. This enhances the insulin secretion induced by ingestion of nutrients [31]. Moreover, inhibition of glucagon and an increase in satiety is seen [23]. The effect on satiety is thought to be a result of stimulation of GLP-1Rs in the hypothalamic satiety centres responsible for regulating appetite [32]. Furthermore, a reduction in appetite is observed due to the inhibition of gastric emptying, which is followed by slower gastrointestinal motility [32]. GLP-1-RAs thus have the benefit of contributing to weight loss [23]. Generally, greater reductions in HbA_1c_ levels and fasting glucose levels are seen in treatment with long-acting GLP-1-RAs compared to short-acting GLP-1-RAs. However, postprandial levels of glucose are seen to be lower in treatment with short-acting GLP-1RAs compared to long-acting GLP-1-RAs [33]. GLP-1-RAs are associated with minimal risk for hypoglycaemia [33]. As for semaglutide and liraglutide, specific effects on appetite centres in the hindbrain have been suggested [34], making it perhaps ideal in combination with TRE. In any case, perceived risk of hypoglycaemia is low.

#### 3.3.3. DPP-4 Inhibitors

DPP-4 inhibitors are competitive reversible inhibitors with high affinity for DPP-4 [35]. DPP-4 is the enzyme that initially cleaves GLP-1, leading to the loss of its insulinotropic action [35]. The effect of the DPP-4 inhibitors on glycaemic control is thought to be largely mediated by GLP-1, but as the glucose-lowering actions of DPP-4 inhibitors are also seen in the absence of GLP-1, one or more other mediators must be present [36]. All DPP-4 inhibitors have the same mode of action; however, there is variation in their pharmacokinetic and pharmacodynamic profiles [37]. The range of bioavailability varies from 30–87% and food intake does not seem to be of significant influence [37]. Due to variation in half-life, some DPP-4 inhibitors should be taken once daily while others should be taken twice a day [37]. As risk of hypoglycaemia is low, the combination with TRE should be unproblematic.

#### 3.3.4. SGLT-2 Inhibitors

In the kidneys of a healthy person, almost all filtered glucose is reabsorbed. The transporter mainly responsible for this is the sodium glucose co-transporter 2 (SGLT-2) which is located in the proximal tubule of the nephron [38]. SGLT-2 inhibitors promote glucosuria, which in turn leads to a direct lowering of blood glucose levels [39]. By promoting osmotic diuresis, the SGLT-2 inhibitors additionally lower blood pressure [40]. The excretion of glucose has also been shown to result in weight loss, mainly by a reduction in fat mass [39]. The risk of hypoglycaemia is low as the effect of SGLT-2 inhibitors does not depend on insulin [39]. Canagliflozin, dapagliflozin, and empagliflozin, representing different SGLT-2 inhibitors, have bioavailability ranging from 65 to 90% and similar half-life times of 10–13 h [38]. By itself, SGLT-2 inhibitors are associated with a low risk of hypoglycaemia and should be ideal for combination with TRE.

#### 3.3.5. Sulfonylureas

Sulfonylureas work directly on pancreatic β-cells by targeting the ATP-sensitive potassium channel [41]. Sulfonylureas have different pharmacokinetic profiles reaching peak plasma concentration within 1.5–4 h. The half-life is less than 10 h for some drugs and more than 24 h for others [31]. However, the biological effects of sulfonylureas often last longer than what could be expected when looking at their half-life. This is due to the formation of active metabolites and receptor interaction [42]. It is recommended to take sulfonylureas 30 min before meals as food can reduce the absorption in the intestines [42]. The dosage varies between the different sulfonylureas as do their rate of absorption, elimination route, and binding site on the target receptor [42]. Improvement of glycaemic control obtained by treatment with sulfonylureas is associated with weight gain [43]. As sulfonylureas confer a risk of hypoglycaemia, combination with TRE should be conducted with caution, perhaps advising more frequent blood glucose measurements to monitor the effect of the combined efforts.

#### 3.3.6. Basal Insulin

Basal insulin analogs are used in order to mimic the actions of the endogenous basal insulin, thus aiming to control the levels of blood glucose in the fasted state [44]. Two generations of long-acting basal insulin analogs are available for treatment [45]. The second generation of long-acting insulins have reduced intraindividual and interindividual variability in treatment response in comparison to the first generation [46]. Moreover, the second generation does not have a peak of action. This results in a minimised risk of hypoglycaemia [46]. However, the risk of hypoglycaemia is higher for the intermediate-acting insulin analogs than for the longer-acting insulin analogs [23]. In addition, treatment with premixed or rapid-acting insulins increases the risk of hypoglycaemia [23]. Therefore, this is a treatment group with theoretical need for caution and dose adjustment when engaging in TRE, although very little clinical experience has accumulated so far.

## 4. Discussion

The systematic search conducted in PubMed identified only four studies investigating TRE in people with type 2 diabetes. In the pilot study by Arnason et al. [19], where all participants were treated with metformin, no hypoglycaemic events were detected during the intervention and the study did not report on any other adverse events. Only one participant was treated with sulfonylurea and one was treated with another unspecified antidiabetic drug. The small sample size, lack of control group, short intervention, and uneven distribution between men (*n* = 1) and women (*n* = 9) are limitations to the study affecting the generalisability to a more complex group of people with type 2 diabetes treated with a wider range of antidiabetic drugs [19]. In the study by Parr et al. [17], which was slightly larger (*n* = 19), participants were treated with either metformin, DPP4-inhibitors, SGLT-2 inhibitors, or did not receive any antidiabetic treatment at all. Three participants were treated with two antidiabetic drugs [17]. Thus, the study investigated TRE in a homogenous group of people taking antidiabetic drugs with low potential of causing hypoglycaemia. Participants did not report any hypoglycaemic events; however, it is uncertain whether instructions to measure glucose levels were given and whether systematic questioning of participants regarding symptoms of hypoglycaemia was conducted. Moreover, participants were only asked to adhere to the eating regimen on as many days as possible (average adherence approx. 5 days per week). It should be considered if participants might have chosen not to adhere to the eating regimen on days where they were at higher risk of experiencing hypoglycaemia, such as days with more extensive exercise. In relation to safety, this study indicates that a 9 h TRE regimen is safe for this group of people with type 2 diabetes for at least 5 days per week.

In the cross-over study by Kahleova et al. [18], participants were instructed to follow a 12-week TRE regimen of 2 meals per day while consuming an energy-restricted diet (~500 kcal/day energy deficit). The 54 participants in that study were treated with a wider range of antidiabetic drugs than in the two studies on TRE in people with type 2 diabetes mentioned above [17,19]. However, participants treated with insulin were not included, which again leads to decreased generalisability of the study. All participants were asked to continue their medication regimen unless experiencing hypoglycaemia repeatedly. In this case, a reduction in medication was to be made by a study physician. Moreover, a glucometer and instructions on how to use it were given to all participants [18]. Despite these measures, there were no reports of any hypoglycaemic events or other adverse events or reductions in use of medicine.

The most recent RCT by Che et al. [20] observed a modest weight loss (3 kg) and improvements in HbA_1c_, insulin resistance and self-rated quality of life as well as reductions in use of antidiabetic medication in response to 12 weeks of 10 h TRE. However, at baseline, participants had only moderate overweight (BMI: 26 kg/m^2^) and a relatively short diabetes duration (5 years) and one could expect greater effects among people with a greater degree of adiposity and metabolic dysfunction. Importantly, no adverse events including hypoglycaemic events were reported and no participants dropped out of the study due to the TRE intervention. Furthermore, on average, participants adhered to their intervention ≥ 6 days/week. The findings suggest that 12 weeks of 10 h TRE is safe, effective, and feasible in people with type 2 diabetes.

Three of the four studies included a small number of participants and intervention durations were short, ranging from 2 to 12 weeks. Therefore, studies of longer duration are needed to assess long-term safety of TRE among people with type 2 diabetes. Additionally, the studies conducted so far included homogenous study populations consisting of only well-regulated people with type 2 diabetes. In order to recommend TRE to people with type 2 diabetes, studies establishing efficacy are needed. Studies should further investigate optimal eating windows, taking efficacy, safety, and feasibility into account before any recommendations can be made. Prior to undertaking fasting regimens, it is advisable for people with type 2 diabetes to consult with their physician to obtain individualised recommendations. Thus, limited conclusions can be made based on the single studies. However, together, the studies could indicate that safety might not be an issue for people with well-regulated type 2 diabetes participating in TRE regimens, as no hypoglycaemic or other adverse events were reported in any of the studies. However, it was unclear how well adverse events were reported and monitored in the studies. More studies which systematically monitor hypoglycaemic events are needed because of the severity of this condition in people with type 2 diabetes. The generalisability of the studies might be limited as the groups of people with type 2 diabetes in these studies were quite homogeneous regarding age, use of antidiabetic drugs, and how well-regulated they were. The RCT by Che et al. [20] included a larger sample size (*n* = 120) but there is a need for larger studies including more heterogeneous groups of people in respect to age, sex, duration, and regulation of type 2 diabetes to investigate the general safety of TRE in a broader group of people with type 2 diabetes.

The most widely used antidiabetic drugs in Denmark in the period 2010–2019 were metformin, insulin, GLP-1-RAs, DPP-4 inhibitors, SGLT-2 inhibitors, and sulfonylureas. With regards to safety, the risk of hypoglycaemia seems to be very low for many of these drugs as described in the results. The risk of hypoglycaemia is higher in patients treated with sulfonylureas and insulin than for other antidiabetic drugs [47]. The low risk of causing hypoglycaemia for metformin, DPP-4 inhibitors, GLP-1-Ras, and SGLT-2 inhibitors indicates that these antidiabetic drugs might be used for treatment of people with type 2 diabetes undertaking TRE regimens without major safety concerns. Insulin and sulfonylureas have higher risk of causing hypoglycaemia. Thus, it might be meaningful to take further precautions before including people with type 2 diabetes taking these antidiabetic agents in TRE regimens. Our data showed that the use of sulfonylureas has declined in the past years, while the use of insulin has slightly increased. It should be mentioned that the graph represents the total prescription sales data in Denmark and thus it does not consider that some of the drugs may be used for other indications than type 2 diabetes, e.g., the 28,000 people with type 1 diabetes in Denmark [48]. Due to global differences in pricing, availability, and the impact of different reimbursement systems, there is a great variation in prescription patterns for treatment of type 2 diabetes. This may affect the generalisability of our findings on use of medication in the present review. Since sulfonylureas and isophane insulin are still a major part of treatment in many countries, and since these are associated with higher risk of hypoglycaemia, implementation of TRE among people treated with these drugs should be carefully monitored. Due to the limited research performed, it could take years before guidelines for people with type 2 diabetes undertaking a TRE regimen can be developed. At that point, there might be a very limited number of people treated with sulfonylureas if the usage continues its decline. Therefore, it might be more important for future studies to focus on drugs more commonly used to treat type 2 diabetes.

The optimal intermittent fasting regimen for people with type 2 diabetes is still unknown and an adjustment protocol for medicine has not been developed. It is therefore suggested that people with type 2 diabetes should not undertake an intermittent fasting regimen without talking to their physician [14]. Some regimens might be associated with higher risk of hypoglycaemia than others. As an example, a randomised parallel group interventional trial of 5/2 intermittent fasting concluded that even with reductions in antidiabetic medication, an increase in the rate of hypoglycaemia was seen [49]. It could be speculated that the length of the fasting period correlates to the risk of hypoglycaemia. Thus, a higher risk of hypoglycaemia can be expected when prolonging the fast and shortening the eating windows of TRE regimens. Based on TRE studies in people with overweight, there is currently no consensus regarding the optimal length of the eating window, but 8–10 h/day have shown promising effects on weight loss and cardiometabolic risk factors, with comparable effects to shorter eating windows, and this allows for proper compliance [7].

In this review, a list of ongoing studies investigating TRE in people with type 2 diabetes is included (Table 2). The studies aim to provide knowledge on the effects of TRE in the management of type 2 diabetes. It has been suggested that TRE may lead to improvement in health regardless of a reduction in weight [12]. However, results from the upcoming studies investigating TRE in people with type 2 diabetes are needed before any firm conclusions with regards to efficacy and safety can be made. Not all the ongoing or planned studies include outcomes related to safety and the studies vary greatly in terms of inclusion and exclusion criteria regarding type of medication. Some of the drugs administered to the participants may not present major concerns with regards to risk of hypoglycaemia, but other potential safety issues should be considered. The main side effects of metformin are related to the gastrointestinal tract and thus metformin is to be administered together with food twice daily [23]. A narrow eating window of a few hours may or may not present a problem for this as metformin would have to be taken fewer hours apart.

## 5. Conclusions

In conclusion, the four studies on TRE included in this review suggest that it might be safe for people with type 2 diabetes to undertake a TRE regimen. However, to make any firm recommendations regarding the safety of TRE in type 2 diabetes, further investigations and larger studies of longer duration are needed.

## Figures and Tables

**Table 1:** Studies investigating time-restricted eating in people with type 2 diabetes.

Study	Country	Study-Design	Intervention Eating Window	Duration of Intervention	Number of Participants	Male M, Female F	Mean Age, Years	Participants Treated with Antidiabetic Drugs	Incidences of Hypoglycaemia or Adverse Events
Arnason et al. (2017) [19]	Canada	Non-randomised Study; 2-week baseline, 2-week intervention, and 2-week follow up.	Daily eating window of 4–6 h	2 weeks	10	M 1 F 9	53.8	Metformin (10) Sulfonylureas (1) Other diabetic medications (1)	No hypoglycaemic events Does not report on other adverse events
Kahleova et al. (2014) [18]	Czech Republic	Randomised crossover study; 2 meals a day for 12 weeks, 6 meals a day for 12 weeks.	Both study arms: (resting energy expenditure × 1.5)−500 kcal/day 2 meals a day (1st meal 6–10 a.m. and 2nd meal 12–4 p.m.) OR 6 meals a day Breakfast, lunch and dinner + 3 smaller snacks in between.	2 × 12 weeks	54	M 29 F 25	59.4	Metformin (41) Sulfonylureas (16) Thiazolidinedione (3) Glinides (2) Acarbose (1) DPP-4 inhibitors (19)	Does not report on hypoglycaemic events No other adverse events
Parr et al. (2020) [17]	Australia	Pre-post, non-randomised. 2-week baseline, 4-week intervention.	Daily eating window between 10 a.m. and 7 p.m.	4 weeks	19	M 9 F 10	50.2	Metformin (10) SGLT-2 inhibitors (3) DPP-4 inhibitors (2)	No hypoglycaemic or other adverse events
Che et al. (2021) [20]	China	Randomised controlled trial	TRE: ad libitum intake: 8 a.m.–6 p.m. CON: Habitual diet	12 weeks	120	TRE: M 31 F 29 CON: M 34 F 26	TRE: 48.2 CON: 48.8	OHA (not specified) TRE: 42 (70%) CON: 46 (77%) Insulin TRE: 19 (32%) CON: 15 (25%)	No adverse events including hypoglycaemic events in TRE group. One hypoglycaemic event in CON.

CON, control group; DPP-4, dipeptidyl peptidase-4; GLP-1-RAs, glucagon-like peptide-1 receptor agonists; OHA, oral hypoglycaemic agents; SGLT-2, sodium-glucose cotransporter-2; TRE, time-restricted eating.

**Table 2:** Ongoing studies investigating time-restricted eating in people with type 2 diabetes registered on ClinicalTrials.gov, accessed on 21 December 2021.

Name of Study	Country	Number of Participants	Intervention	Glucose Levels and Antidiabetic Medication	Estimated Timeline	Outcomes
Effect of Time-restricted Eating on Blood Glucose and Behavior in Patients With Type 2 Diabetes Mellitus (TREAT BB)	China	35	12 weeks intervention with daily 10 h eating window	HbA_1c_ 7.0–8.5% No use of insulin, long-acting insulin secretagogues, GLP-1 receptor agonist, DPP-4 inhibitor, SGLT-2 inhibitor	Start: 15 April 2021 Primary completion date: April 2022 Study completion Date: April 2022	Adverse events (not specified) HbA_1c_ and mean glucose and time in range measured using CGM
Application of Time Restriction Feeding in Patients With Type 2 Diabetes Mellitus	USA	30	1 week with daily 12 h eating window	HbA_1c_ ≥ 8% Stable anti-diabetic medication	Start: October 2021 Primary completion date: July 2023 Study completion Date: July 2024	No outcomes regarding drugs, hypoglycaemia, or adverse events Mean glucose (unspecified method) HbA_1c_
Effect of Eating Within a Limited Time on Sugar Sensitivity and Liver Sugar Stores of People With Type 2 Diabetes.	The Netherlands	21	3 weeks intervention with daily 10 h eating window	Non-insulin-treated type 2 diabetes No use of SGLT-2 inhibitors or insulin	Start: 31 January 2019 Primary completion date: 3 February 2021 Study completion Date: 3 February 2021	No outcomes regarding drugs, hypoglycaemia, or adverse events Insulin sensitivity measured by hyperinsulinemic clamp
A Comparison Between the Effects of Conventional Diets vs Intermittent Fasting Diabetic and Pre-diabetic Patients	Pakistan	128	12 weeks intervention: (1) calorie restriction, (2) TRE with daily 8 h eating window, (3) calorie restriction and TRE with daily 8 h eating window	Glycaemic values belonging to diabetes or prediabetes category (not specified) No use of insulin or sulfonylureas	Start: 1 September 2020 Primary completion date: 15 May 2021 Study completion Date: 15 May 2021	No outcomes regarding drugs, hypoglycaemia, or adverse events Fasting glucose, HbA_1c_, OGTT
TREAT to Improve Cardiometabolic Health (NY-TREAT)	USA	52	12 months intervention with daily ≤10 h eating window	Prediabetes and/or fasting glucose ≥ 100 mg/dL and/or HbA_1c_ 5.7% or type 2 diabetes diet-controlled and/or treated with metformin and meeting 2 or more of the metabolic syndrome criteria	Start: 26 May 2021 Primary completion date: 31 January 2025 Study completion Date: 30 June 2025	No outcomes regarding drugs, hypoglycaemia, or adverse events Matsuda index Insulinogenic index
Time Restricted Eating As Treatment (TREAT) for Diabetes Mellitus: A Pre-Post 12 Week Study on the Effectiveness of Intermittent Fasting in Asians With Type 2 Diabetes Mellitus	Singapore	50	12 week intervention with daily 8 h eating window	Newly diagnosed T2D Solely dietary control	Start date: 14 January 2019 Primary completion date: 30 June 2020 Study completion Date: 30 June 2020	No outcomes regarding drugs, hypoglycaemia, or adverse events
TREAT (Time Restricted EATing) to Improve Cardiometabolic Health	USA	52	Followed up to 12 months. 10 h eating window a day	Prediabetic (ADA criteria 2019) OR T2D solely diet-controlled, and/or treated with metformin and HbA_1c_ ≤ 7%	Start date: November 2020 Primary completion date: December 2024 Study completion date: September 2025	No outcomes regarding drugs, hypoglycaemia, or adverse events
Time Limited Eating in Adolescents With Type 2 Diabetes	USA	40	12 weeks intervention with daily 8 h eating window for 5 days per week	T2D and HbA_1c_ < 9% Monotherapy with metformin	Start date: 1 January 2021 Primary completion date: 1 December 2024 Study completion date: 1 December 2026	No outcomes regarding drugs, hypoglycaemia, or adverse events
Effect of Time Restricted Feeding on Hepatic Glycogen Depletion and Insulin Sensitivity in Adults With Type 2 Diabetes	The Netherlands	34	Randomised controlled cross-over design with two 3 weeks arms and a 4 weeks wash-out period. Daily 10 h eating window.	T2D No use of SGLT-2 inhibitors or insulin	Study start date: 31 January 2019 Primary completion date: October 2019 Study completion date: December 2019	No outcomes regarding drugs, hypoglycaemia, or adverse events
Time-Restricted Feeding on Glucose Homeostasis and Quality of Life	Kuwait	50	12 weeks intervention Daily 6 h eating window	T2D with HbA_1c_ 6.5–12% Any diabetes medication	Study start date: 10 July 2019 Primary completion date: 1 March 2020 Study completion date: 1 March 2020	Secondary outcome: Change in diabetes medications between the intervention and control arms
Using Early Time Restricted Feeding and Timed Light Therapy to Improve Glycaemic Control in Adults With Type 2 Diabetes	USA	344	16 weeks intervention 8 h daily eating window	HbA_1c_ 7.0–10.0% and a change of less than 0.7% 6 months prior to study Treatment with metformin, sulfonylureas, DPP-4 inhibitors, and/or GLP-1-RAs on a stable dose for a minimum of 6 months or no use of antidiabetics.	Study start date: October 2020 Primary completion date: August 2023 Completion date: August 2023	24 h glucose levels (Time Frame: 16 weeks) Time-weighted mean, fasting, peak, standard deviation, and excursion (maximum–minimum) values (mg/dL)
The Impact of Time Restricted Feeding (TRF) in Improving the Health of Patients With Metabolic Syndrome	USA	35	12 weeks intervention 10 h eating window a day	Elevated fasting glucose ≥ 100 mg/dL or drug treatment of elevated blood glucose No use of medication with known effect on appetite	Study start date: 28 July 2017 Primary completion date: 31 January 2019 Study completion date: June 2020	Mean blood glucose (Time Frame: 12 weeks) Measured using CGM
Feasibility Study of a Low-Carb/Time-restricted Feeding Protocol in Insulin-Using Type 2 Diabetics	USA	20	6 months intervention Low carbohydrate diet (30–60 g) 8 h eating window with 2 meals daily	T2D Use of basal insulin glargine or detemir. Stable T2D regimen for > 3 months and HbA_1c_ 7–10%	Study start date: October 2020 Primary completion date: October 2021 Study completion date: October 2021	Effectiveness of intervention: Changes in insulin dosage
Therapeutic Effects of Time Restricted Feeding and Calorie Restriction in Patients With Prediabetes and Diabetes	Pakistan	250	12 weeks intervention Intervention groups of: TRE (8 h eating window a day), CR (deficit of 500 calories), and combined TRE and CR	TDM or prediabetic No use of insulin or sulfonylureas	Study Start Date: September 2020 Primary completion date: January 2021 Study completion date: February 2021	No outcomes regarding drugs, hypoglycaemia, or adverse events

ADA, American Diabetes Association; CGM, continuous glucose monitoring; CR, calorie restriction; DPP-4, dipeptidyl peptidase-4; GLP-1-RAs, glucagon-like peptide-1 receptor agonists; OGTT, oral glucose tolerance test; SGLT-2, sodium-glucose cotransporter-2; TDM, treated diabetes mellitus; TRE, time-restricted eating; TRF, time-restricted feeding; T2D, type 2 diabetes.

## Data Availability

Not applicable.
